# *In Vivo* Cell Reprogramming towards Pluripotency by Virus-Free Overexpression of Defined Factors

**DOI:** 10.1371/journal.pone.0054754

**Published:** 2013-01-23

**Authors:** Açelya Yilmazer, Irene de Lázaro, Cyrill Bussy, Kostas Kostarelos

**Affiliations:** Nanomedicine Lab, UCL School of Pharmacy, UCL School of Life & Medical Sciences, University College London, Brunswick Square, London, United Kingdom; Justus-Liebig-University Giessen, Germany

## Abstract

The ability to induce the reprogramming of somatic mammalian cells to a pluripotent state by the forced expression of specific transcription factors has helped redefine the rules of cell fate and plasticity, as well as open possibilities for disease modeling, drug screening and regenerative medicine. Here, we hypothesized that the non-viral forced expression of the four originally discovered defined factors (OKSM) in adult mice could result in *in vivo* reprogramming of cells in the transfected tissue *in situ*. We show that a single hydrodynamic tail-vein (HTV) injection of two plasmids encoding for *Oct3/4, Sox2, Klf4* and *c-Myc* respectively, are highly expressed in the liver tissue of Balb/C adult mice. Hallmark pluripotency markers were upregulated within 24–48 h after injection, followed by down-regulation of all major hepatocellular markers. Generation of transcriptionally reprogrammed cells *in vivo* was further confirmed by positive staining of liver tissue sections for all major pluripotency markers in Balb/C mice and the Nanog-GFP reporter transgenic strain (TNG-A) with concomitant upregulation of GFP expression *in situ*. No signs of physiological or anatomical abnormalities or teratoma formation were observed in the liver examined up to 120 days. These findings indicate that virus-free expression of OKSM factors *in vivo* can transcriptionally reprogram cells *in situ* rapidly, efficiently and transiently, absent of host tissue damage or teratoma formation.

## Introduction

Forced reprogramming of somatic cells into a pluripotent, stem cell-like state by the ectopic expression of specific transcription factors results in the generation of induced pluripotent stem (iPS) cells. Such transcription factor cell reprogramming has been achieved today by viral [1], [2], [3] and non-viral [4], [5], [6], [7] gene transfer, protein cytoplasmic translocation [8], [9], miRNA [10] and is changing the landscape in developmental biology, can potentially resolve all ethical concerns about the use of embryonic stem cells and open further opportunities for regenerative medicine. The original discovery by Yamanaka and colleagues that the *in vitro* expression of four transcription factors, Oct3/4, Klf4, Sox2, c-Myc (OKSM) was capable to revert fully differentiated mouse and human skin fibroblasts into iPS cells [1], [11] constitutes the most widely used transcription-based reprogramming technology today.

The initial reports of transcription-mediated somatic cell reprogramming involved the use of retroviruses to stably transduce skin fibroblasts with defined transcription factors [1], [2], [11]. This methodology of gene transfer is still today the most popular way to reprogram animal and human somatic cells despite the risks from insertional mutagenesis, stable transduction and long-term gene expression of known proto-oncogenes [12], [13]. Moreover, the vast majority of current methodologies to generate iPS cells involve use of long-term culture conditions and treatment of cells with multiple rounds of gene transfer vectors, growth factors, antibiotics and other cell media cocktails to promote reprogramming and select for pluripotency. All of these are considered major culprits for the potential risks associated with the ensuing cells as recent studies investigating the genomic integrity of iPS have alluded to [14], [15], [16]. In terms of iPS generation using non-viral gene transfer vectors, plasmid DNA [4], [5], [6] or RNA [7], [10] delivery using liposomes or electroporation have been reported. Compared to viruses, episomal vectors are generally considered safer, however transduction and reprogramming efficiencies are much lower [13]. Alternatively, Warren *et al.* reported somatic cell reprogramming *in vitro* by direct delivery of synthetic mRNAs [7]. Although this methodology offers significantly higher reprogramming efficiency, high RNA dosages, multiple rounds of transfection and complex cell culturing protocols are still needed [13].

Due to the paradigm-shifting nature of transcription-induced reprogramming to pluripotency there is still limited understanding of the exact mechanisms and pathways implicated in induced cell reprogramming, and the exact features of reprogrammed cells [17], [18]. Morever, the majority of experimental evidence today is based on the concept of extraction and *in vitro* manipulation of the somatic cells to be reprogrammed, leading to the array of caveats mentioned above that make clinical translation of iPS cells seem distant [19], [20], [21]. In the present work, we hypothesized that *in vivo* forced expression of the OKSM transcription factors by non-viral transient over-expression within living tissue can induce cell reprogramming towards pluripotency. In order to test this hypothesis we chose the most naive, non-viral gene transfer technology available today: large-volume, rapid hydrodynamic tail vein (HTV) injection of plasmid DNA [22], [23] encoding the originally proposed OKSM factors. This gene transfer methodology circumvents most complications or risks associated with viral gene transfer vectors, as has been previously shown in numerous preclinical [24], [25] and clinical [26], [27] studies allowing unprecedented levels of exogenous gene expression in hepatocytes.

## Methods

### Plasmids

Reprogramming plasmids pCX-OKS-2A encoding *OCT3/4, KLF4, SOX2*; pCX-cMyc encoding *CMYC* and pCAG-GFP encoding *eGFP* under the control of CAG promoter (as previously described by Okita et al. [4]) were obtained from Addgene (USA) as bacterial stabs. Research grade plasmid production was performed from these stabs (Plasmid Factory, Germany). pGFP-Luc plasmid (Clontech, USA) encodes for the *eGFP* and *Luc* transgenes under the control of a CMV promoter. Vector maps of all plasmids used in this work are included in **Figure S5**.

### Animals and Hydrodynamic Tail Vein (HTV) Injection of Plasmids

All experiments were performed with prior approval from the UK Home Office under a Home Office project license (PPL 80/2296). Female Balb/C mice, 6 weeks old were purchased from Harlan, UK. TNG-A mice which carry the eGFP reporter inserted into the Nanog locus [28], were a kind gift from The Wellcome Trust Centre for Stem Cell Research, University of Cambridge, UK. TNG-A mice and WT controls, were of 129 background and were bred and genotyped at the UCL. Mice were allowed one week to acclimatize prior to use. Mice (4 animals/group)were warmed in a 37°C heating chamber, anesthetized with isofluorane and were injected via tail vein in 5–7 sec with 1.5 ml of 0.9% saline including 75 µg of pCX-OKS-2A with and without 75 µg of pCX-cMyc or 150 µg pCAG-GFP plasmids or no plasmid. Mice were culled at different time points, such as 2, 4, 8, 12, 24, 50, 120 days after HTV injections.

### Isolation of Hepatocyte Population

Mice livers were perfused as previously described [36] with some modifications. In brief, livers were first perfused with Ca^2+^ and Mg^2+^ free Hank’s buffered salt solution (Sigma-Aldrich, UK) and then with liver digest medium (Gibco, UK) at 37°C. After digestion, liver was washed with Hepatocyte Wash Medium (HWM, Gibco, UK) and cell suspension was passed through a 100 µl cell strainer (VWR, UK) at 4°C. Cells were centrifuged at 50 g for 5 min to separate parenchymal cells (PC including hepatocytes) which were collected in pellet and non-parenchymal cells (NPC including Kupffer cells and epithelial cells) which stayed in supernatant. The hepatocyte fraction was collected after washing twice with HWM.

### RNA Isolation and Reverse Transcription-quantitative PCR (RT-qPCR) Analysis

Following liver perfusion and isolation of hepatocytes, RNeasy Blood and Tissue Kit (Qiagen, UK) were used to isolate total RNA. cDNA synthesis from 1 µg of RNA sample was performed by iScript cDNA synthesis kit (Bio-Rad, UK) according to manufacturer’s instructions. Two microliters of each cDNA sample was used to perform RT-qPCR reactions with iO SYBR Green Supermix (Bio-Rad, UK). Primer sequences are shown in Table S1. Samples were run on CFX-96 Real Time System (Bio-Rad, UK) with the following protocol: 95°C for 3 min, 1 cycle; 95°C for 10 sec, 60°C for 30 sec, – repeated for 40 cycles. *β-actin* was used as a housekeeping gene and gene expression levels were normalized to saline groups.

### Flow Cytometry

Cell density was adjusted to 1×10^7^ cells/ml and 100 µl suspensions were aliquoted in microfuge tubes. BD Mouse Pluripotent Stem Cell Transcription Factor Analysis Kit (BD Biosciences, UK) were used to analyze OCT3/4-positive or NANOG-positive cells. In brief, cells were firstly fixed with BD Cytofix fixation buffer and permeabilized with 1X BD Perm/Wash buffer. Then cells were incubated with either anti-mouse OCT4-PerCP-Cy5.5 or anti-mouse NANOG-PE for 30 min according to manufacturer’s instructions. Negative and isotype controls were included in each experiment. Stained cells were then analyzed by CyAn™ ADP High-Performance Research Flow Cytometer (DakoCytomation, USA) at the Institute of Child Health, University College London, UK.

### Quantification of Serum Cytokine Levels

Blood was collected from mice at different time points. Serum levels of ALT, AST, ALP, GLDH were determined by Diagnostic Laboratories in the Royal Veterinary College, London, UK.

### Immuno-histochemistry (IHC) of Mouse Livers

Livers were perfused with 10 mL HBSS, pre-warmed at 37°C, then immediately immersed into isopentane, pre-cooled in liquid nitrogen or into stabilized 4% paraformaldehyde for fixation. Frozen livers were stored at −80°C until further processing. Fourteen micron thick sections were prepared on a Cryomicrotome (Leica Microsystems, CM3050S), air-dried for 1hour at room temperature, before storage at −20°C. Before staining, liver sections were post-fixed with methanol, pre-cooled at −20°C, for 10 min at −20°C, then air-dried for 15 min and finally washed twice with PBS for 5 min. For Oct4, Sox2 and Nanog, we used a conventional immunostaining protocol that consisted of 1 h incubation in blocking buffer (5% goat serum-0.1% Triton in PBS pH 7.3) at room temperature, followed by two washing steps with PBS (1%BSA- 0.1% Triton, pH 7.3) before overnight incubation at +4°C with the different primary antibodies [(rabbit pAb anti-OCT4 (ab19857,3 µg/ml, Abcam, UK)/rabbit pAb anti-SOX2 (ab97959, 1 µg/ml, Abcam, UK)/rabbit polyclonal anti-NANOG (ab80892, 1 µg/ml, Abcam, UK]). Next day sections were washed (2 min each) with PBS and incubated (1.5 hours at room temperature) with the secondary antibody (goat polyclonal anti-rabbit IgG labeled with Cy3, 1/250, Jackson ImmunoResearch Laboratories Inc.). For SSEA1 immunostain, a mouse monoclonal antibody (ab16285, 20 µg/ml, Abcam,UK) was used, which make it more suitable to use a specific immunodetection kit to localize mouse antibodies on mouse tissues (vector MOM immunodection kit, Vector Laboratories). The endogenous liver avidin/biotin were blocked with Avidin/blocking Kit (Vector Laboratories, UK). The staining procedure was performed according to the supplier recommendations, using either the provided secondary biotinylated anti-mouse IgG revealed by fluorescein-tagged Avidin (1/250, Vector Laboratories) or a goat polyclonal anti-mouse IgG labeled with Cy3 (1/250, Jackson ImmunoResearch Laboratories Inc.). Then liver sections were washed with PBS and mounted in DAPI and antifade containing medium (Vectashield mounting medium, Vector Laboratories, UK). Slides were visualised under epi-fluorescence microscope (Zeiss Axio Observer). Paraformaldehyde fixed samples were paraffin-embedded and liver sections were stained with Haematoxylin and Eosin (H&E) by Diagnostic Laboratories in the Royal Veterinary College, London, UK. Random images were captured by light microscopy for different treatment groups.

### Periodic Acid Schiff (PAS) Staining of Liver Sections

Liver samples were fixed in paraformaldehyde, paraffin-embedded and liver sections were stained with PAS stain (Sigma, UK). Random images were captured by light microscopy for saline or OKSM treatment groups.

### Alkaline Phosphatase (ALP) Staining of Cell Cultures and Liver Sections

ALP activity staining was performed using the BCIP/NBT liquid substrate system (Sigma UK). Methanol-fixed cell cultures were first washed with HEPES buffer and then incubated at 37°C for 30 min with the BCIP/NBT liquid substrate system. Color development was stopped by rinsing with water. In order to determine ALP activity in the tissue, frozen liver sections were prepared as described above and incubated with BCIP/NBT liquid substrate system for 30 min. Sections were washed with water and mounted. Random images were captured by light microscopy (10×) for saline, GFP or OKSM treatment groups.

### Statistical Analysis

Experiments were performed with at least four animals per group. Statistical analysis was performed by analysis of variance and Tukey's pairwise comparison using SPSS software, version 16.0.

## Results

Mice were injected by HTV with an equimolar mix of two plasmids, pCX-OKS-2A and pCX-cMyc, encoding for the OKS and M reprogramming factors respectively. HTV injection of plasmid DNA has been known to result in high levels of gene expression in hepatocytes [22], [23] and primary hepatocytes from OKSM-injected animals were extracted at different time points here. RT-qPCR and flow cytometry were used to analyze the expression levels for various reprogramming (*Oct3/4, Sox2, c-Myc*), pluripotency (*Nanog, Ecat1, Rex1, Cripto, Gdf3* and endogenous *Oct3/4, Klf,4* or *Sox2*) and hepatocyte markers (*Alb, Trf, Aat*) directly after extraction of the hepatocytes at different time points after HTV injection.

**Figure 1:**
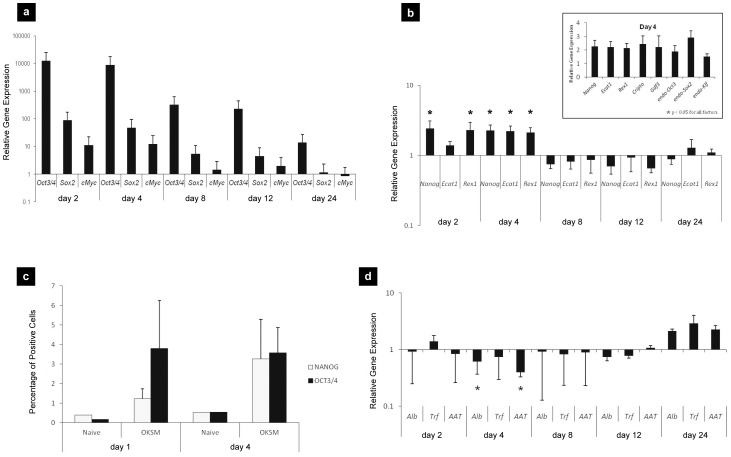
*In vivo* overexpression of OKSM transcription factors in adult mouse liver. Balb/C mice were HTV injected with 0.9% saline alone, 75 µg of pCX-OKS-2A and 75 µg pCX-cMyc in 0.9% saline and at days 2, 4, 8, 12, 24, RT-qPCR analysis of hepatocytes was performed to determine the relative gene expression of: (**a**) transfected transcription factors (OKSM) and (**b**) endogenous pluripotency markers. All gene expression levels were normalized to HTV-injected saline group (*p<0.05 indicates statistically significant differences between the expression levels of pluripotency markers in the OKSM and saline HTV-injected groups, obtained by the analysis of variance and Tukey's pairwise comparison); (**c**) flow cytometry analysis of OCT3/4 positive and NANOG positive cells in liver extracts; (**d**) relative gene expression of hepatocyte markers as determined by RT-qPCR. All gene expression levels were normalized to saline HTV-injected group (* p<0.05 indicates statistically significant differences between the expression levels for hepatocyte markers in the OKSM and saline HTV-injected groups, obtained by the analysis of variance and Tukey's pairwise comparison).

A significant increase in the gene expression of all transduced transcription factors was observed on day 2 that decreased over time **(** **Figure 1a** **)**. At the same time endogenous pluripotency markers were upregulated, reached peak values on day 4 and decreased to background levels from day 8 onward **(** **Figure 1b** **)**. Protein expression in the hepatocyte extracts from the transduced liver tissue using flow cytometry indicated that on day 1 only OCT3/4 was expressed, whereas by day 4 both OCT3/4 and NANOG positive cells were detected **(** **Figure 1c** **)**. Examination of the expression levels of hepatocyte markers by RT-qPCR indicated that on day 2 they were similar to those of animals HTV injected with saline, while on day 4 significant down-regulation in hepatocyte marker expression was considered as another indication of cell reprogramming, manifested by the de-differentiation of hepatocytes **(** **Figure 1d** **)**. Later than day 8, hepatocyte markers returned back to control levels, while on day 24 increased levels of these markers were obtained.

**Figure 2:**
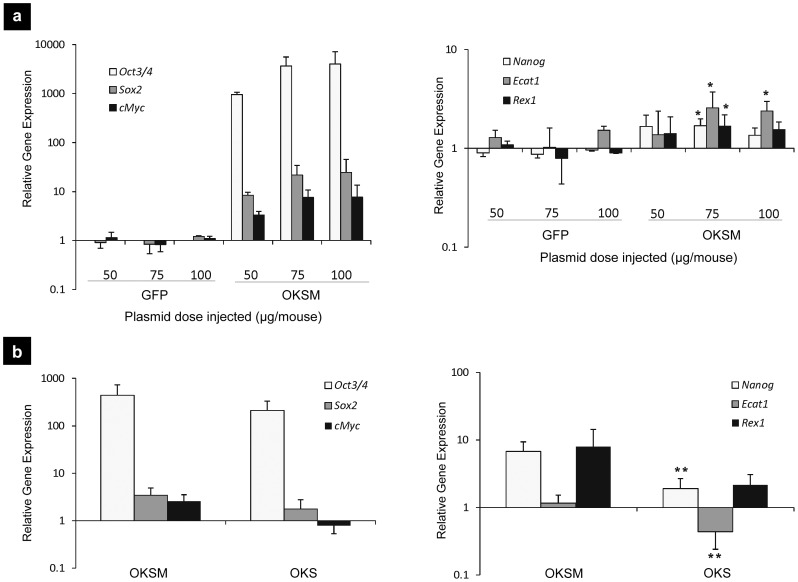
OKSM and OKS factor overexpression with dose-response in adult mouse liver. Balb/C mice HTV injected with 0.9% saline alone, pCX-OKS-2A with (OSKM) and without (OKS) pCX-cMyc in 0.9% saline, or pCAG-GFP in 0.9% saline, at the indicated doses. On day 4, RT-qPCR analysis of hepatocyte extracts was performed. (**a**) Expression levels of the injected reprogramming transcription factors and endogenous pluripotency genes were determined for plasmid dose-escalation (total plasmid dose 50, 75 and 100 µg/animal). All gene expression levels were normalized to the HTV-injected saline group (*p<0.05 indicates statistically significant differences between the expression levels of pluripotency markers in the OKSM and saline HTV-injected groups, obtained by the analysis of variance and Tukey's pairwise comparison); (**b**) Expression levels of the injected reprogramming transcription factors and endogenous pluripotency genes with and without inclusion of *cMyc*. All gene expression levels were normalized to HTV-injected saline group (**p<0.01 indicates statistically significant differences between the expression levels of pluripotency markers in the OKSM and OKS injected groups, obtained by the analysis of variance and Tukey's pairwise comparison).

Transgene expression by HTV injection was monitored separately by injection of control plasmids encoding for GFP-luciferase (pCMV-GFP-luc) and eGFP (pCAG-eGFP). **Figure S1** confirms that hepatocytes were the targeted cell population in the liver following HTV injection as previously reported. The kinetics of transgene expression in the liver following HTV injection with the OKSM plasmids indicated that from day 9 onward, pluripotency factors began to be down-regulated reaching naive levels by day 22 **(Figure S2)**. The effect of plasmid dose was then studied by HTV injections of OKSM against control (pCAG-eGFP) plasmid DNA at an escalated dose regime **(** **Figure 2a** **)**. A sharp increase in the levels of gene expression profile of the transduced reprogramming and other endogenous pluripotent factors on day 4 after administration was observed only in the case of OKSM plasmid DNA injections, and the levels of up-regulation reached a plateau at 75 µg/animal. *c-Myc* is a known oncogene and its use in cell reprogramming (in particular for *in vivo* strategies) is preferable to be avoided. However, a decrease in the efficiency of reprogramming has been previously described in the absence of this factor [30]. The effect of the presence or absence of *c-Myc* in the reprogramming cocktail was also investigated in the present study (**Figure 2b**). *In vivo* cell reprogramming could be achieved without *c-Myc,* however the levels of pluripotency markers Nanog and Ecat1 observed were significantly lower compared to use of the OKSM cocktail, in line with the previous reports omitting *c-Myc* from *in vitro* cell reprogramming.

**Figure 3:**
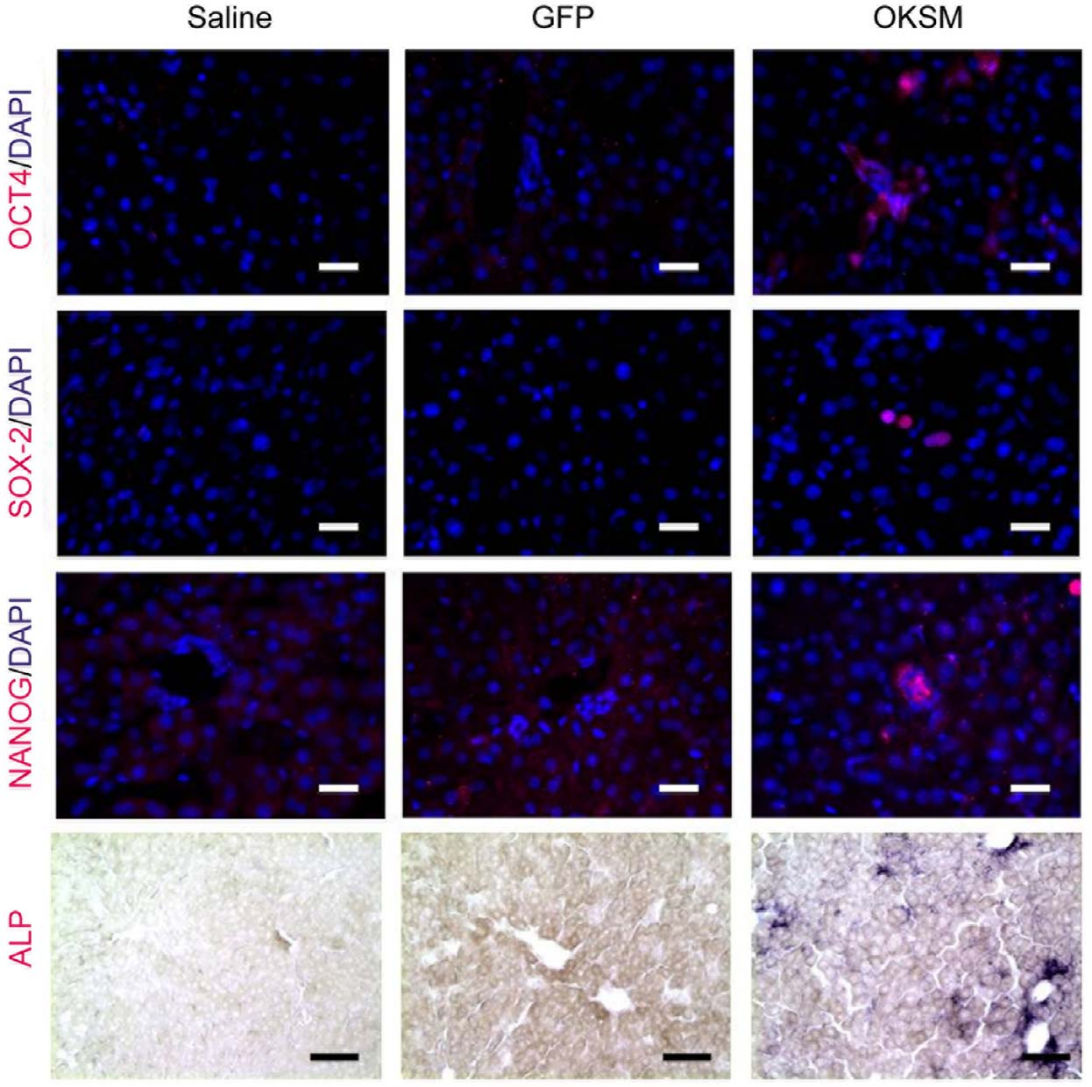
*In vivo* cell reprogramming on adult mouse liver tissue by immunohistochemistry. Balb/C mice HTV injected with 0.9% saline alone, 75 µg of pCX-OKS-2A and 75 µg pCX-cMyc in 0.9% saline, or 150 µg of pCAG-GFP in 0.9% saline and at day 4, livers were collected and frozen tissue sections were stained with anti-OCT4, anti-SOX2 or anti-NANOG antibodies to assess immunoreactivity, or BCIP/NBT to determine ALP activity in the tissue (40x). Scale bars represent 100 µm.

**Figure 4:**
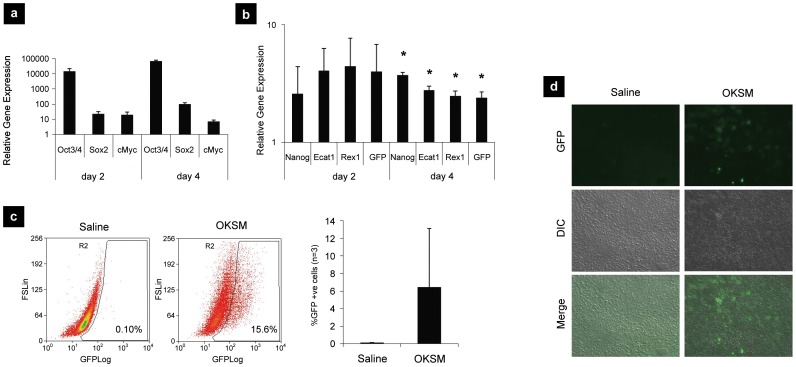
*In vivo* cell reprogramming in TNG-A mice. TNG-A mice were HTV injected with 0.9% saline alone, 75 µg of pCX-OKS-2A and 75 µg pCX-cMyc in 0.9% saline and at days 2, 4, RT-qPCR analysis of hepatocytes was performed to determine the relative gene expression of: (**a**) transfected transcription factors (OKSM) and (**b**) endogenous pluripotency markers. All gene expression levels were normalized to HTV-injected saline group (*p<0.05 indicates statistically significant differences between the expression levels of pluripotency markers in the OKSM and saline HTV-injected groups, obtained by the analysis of variance and Tukey's pairwise comparison); (**c**) flow cytometry analysis of GFP positive cells in liver extracts; (**d**) liver tissue frozen and sectioned to image GFP-positive cells with fluorescence microscopy at day 4 (10x).

To further interrogate the occurrence of *in vivo* induced cell reprogramming in the liver by the forced expression of the OKSM transcription factors, tissue sections from transfected Balb/C mice were directly stained immunohistochemically (IHC) at day 4 (post-HTV) for different pluripotency markers (Oct3/4, Sox2, Nanog and ALP). **Figure 3** shows that distinctive IHC-positive cells were obtained for all four markers only in the case of animals injected with the OKSM plasmids compared to saline or GFP plasmid HTV-injected groups. Positive staining for Nanog was obtained reproducibly in all liver sections in all OKSM-transfected animals indicating the presence of transcriptionally reprogrammed cells throughout the liver tissue (**Figure S3**). Considering the critical role of Nanog in the control of pluripotency, we performed a separate experiment using the transgenic strain TNG-A that carries the eGFP reporter inserted into the Nanog locus [28]. HTV injection of the OKSM plasmids in TNG-A mice showed enhanced gene expression in both reprogramming and endogenous pluripotency markers on days 2 and 4 post-injection **(** **Figure 4a & b** **)**. Hepatocyte extracts from OKSM injected TNG-A mice on day 4 were analyzed by flow cytometry for eGFP protein expression, revealing that 6–15% of the total cell population were eGFP positive **(** **Figure 4c** **)**. Lastly, further confirmation of the generation of reprogrammed cells in the liver was offered by the presence of eGFP-positive cells in frozen tissue sections imaged by fluorescence microscopy **(** **Figure 4d** **)**.

**Figure 5:**
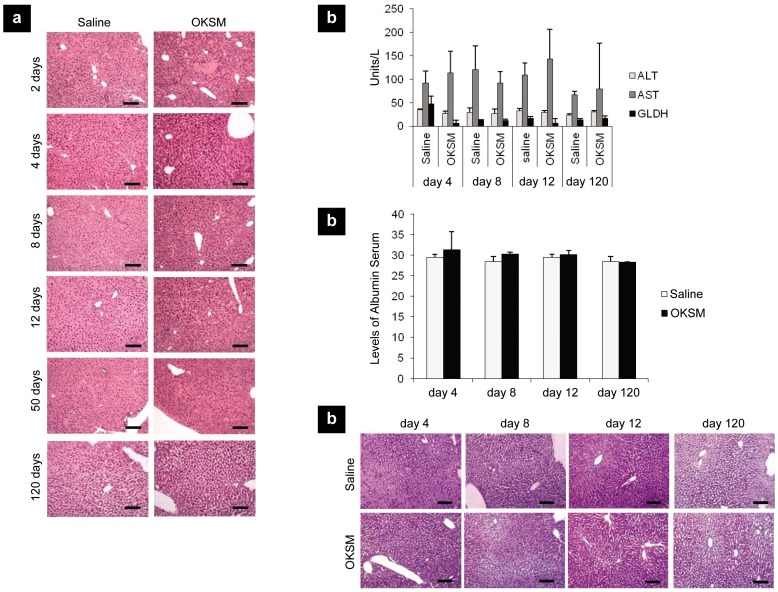
The effect of *in vivo* cell reprogramming on hepatotoxicity and liver damage. Balb/C mice HTV injected with either 75 µg of pCX-OKS-2A and 75 µg pCX-cMyc in 0.9% saline or 0.9% saline only. On days 2, 4, 8, 12, 50 and 120 livers and sera were isolated. (**a**) H&E staining of liver sections; (**b**) levels of liver enzymes and (**c**) albumin were analyzed; (**d**) liver sections were PAS stained to determine glycogen storage levels. Representative images were captured with light microscopy (10x). Scale bars represent 100 µm.

One of the key concerns by induction of reprogramming towards pluripotency *in vivo* may be the spontaneous occurrence of teratomas within the tissues where reprogrammed cells are generated [12], [19]. To address this, animals HTV-injected with the reprogramming factors were kept for a period of 120 days and at frequent intervals (days 2, 4, 8, 12, 50 and 120) different groups were analyzed heamatologically and histologically (**Figure 5a**). HTV injection of plasmid DNA can result in moderate tissue damage manifested by increased serum levels of liver enzymes at early time points [26], which was also observed here at day 2 for both saline and OKSM injected groups (**Figure 5a** **& S4**). H&E staining of liver tissues indicated that HTV injection of OKSM plasmids did not lead to any tissue damage beyond day 2, nor development of teratomas at later time points, with all liver sections and animals exhibiting healthy structural morphology and behavior with no signs of dysplasia (**Figure 5a**). Serum levels were also analyzed for liver enzymes over the same period, with no aberrant change in the levels of ALT, AST and GLDH between the saline-injected and OKSM-injected groups (**Figure 5b**). Albumin levels and glycogen staining of liver sections (**Figure 5c & d**) further confirmed no hepatic structural or functional abnormality throughout the course of the study for any of the animals. These findings in conjunction with the gene and protein expression analyses of the liver tissue (Figure 1) suggested that transcriptional cell reprogramming was occurring *in vivo* rapidly after HTV-injection of the OKSM factors, similar to recent direct reprogramming (transdifferentiation) studies [29], [30], [31]. We speculate that the endogenous tissue milieu (growth factors, homeostatic signaling cues) leads to rapid loss of the transcriptionally reprogrammed cells into phenotypically normal, functioning hepatocytes. However, much more work using lineage-tracing techniques is needed to elucidate the mechanisms and extent of *in vivo* cell reprogramming to a pluripotent state.

## Discussion

This study provides previously unreported evidence that direct *in vivo* transcriptional cell reprogramming towards the pluripotent state in adult mammalian somatic (hepatocyte) cells is possible using non-viral transfection of plasmids encoding the OKSM (or OKS) transcription factors. This occurs following rapid kinetics (within 24–48 h) that are, in principle, reminiscent of the kinetics described for somatic cell reprogramming using the egg and oocyte nuclear transfer methodologies [32]. Zhou et al. have previously reported rapid and highly efficient *in vivo* conversion of pancreatic exocrine cells directly into insulin secreting β-cells without reverting to a pluripotent state (i.e. by transdifferentiation) using adenovirus-mediated transcription factor (not OKSM) overexpression [33]. Very recently, three studies have also offered proof-of-concept evidence that transcriptionally-induced, direct transdifferentiation from fibroblasts to cardiomyocyte-like cells in infracted heart tissue *in situ* can offer therapeutic benefits [29], [30], [31]. In all such previous work direct conversion to different lineages is reported without reprogramming towards a plutipotent state, shown to take place rapidly and efficiently (compared to *in vitro* transdifferentiation methodologies). From our studies herein, *in vivo* cell reprogramming by overexpression of OKSM transcription factors is also shown to be rapid and efficient enough to be detected *in situ*, however transiently maintained. According to our semi-quantitative analysis based on FACS and IHC of liver sections from transfected Balb/C and TNG-A mice, it took place efficiently in the order of 5–15% of the total hepatocyte population. Even though upregulation of eGFP and pluripotency markers in the TNG-A mice strongly suggest that HTV injection of OKSM plasmids can result in adult somatic cell reprogramming toward pluripotency *in vivo*, further studies are needed to determine the level of pluripotency achieved, similar to previous Nanog-activity studies using ES cells [34], as well as more detailed characterization of the extracted reprogrammed cells in relation to the loss of hepatocyte phenotype. Moreover, the *in vivo* cell reprogramming towards pluripotency in fully developed mammals shown here is also in agreement with recently reported cell reprogramming for non-mammalian tissue [35] and did not lead to any structural or functional side effects in the liver, nor did it lead to any manifestation of carcinogenesis or teratoma formation.

Further progress from this work will be the isolation and characterization of the transcriptionally reprogrammed cells from the primary hepatocyte extracts. Determination of the quality and level of functional pluripotent capacity of those cells, their genomic stability and epigenetic character, along with all the comparative studies currently undertaken by numerous laboratories to define the similarities and differences between iPS and ES cells will be needed. Also, the mechanistic basis of direct *in vivo* induced reprogramming of somatic cells to a pluripotent state and the dissection of the reasons behind the rapid kinetics observed, the extent and possible implications of transient cell reprogramming to ground-state pluripotency that could possibly take place transiently *in situ*, and the mechanisms by which the tissue microenvironment drives rapid re-differentiation in the host cells will have to be determined.

Enhancement of the level of *in vivo* cell reprogramming to a pluripotent state can also be envisaged by improvements in gene transfer methodology, dosing regimen and vector construct design. Finally, it can be envisioned that improved (and less invasive) protocols and technologies for the possible generation of *in vivo* induced pluripotent cells may lead to a general methodology for the extraction of autologous cells from tissue (liver or other) rapidly and with minimization of risks (mutagenesis, media contamination, xenobiotic reagents, etc) from culturing conditions and protocols. Overall, this study offers the basis of a rapid, virus-free, efficient, and in the absence of culturing complications *in vivo* platform to study reprogramming mechanisms *in situ* using adult, fully developed, somatic tissue.

## Supporting Information

Figure S1 Transfection after HTV injection. Balb/C mice were HTV injected with pCMV·GFP-Luc in 0.9% saline and hepatocytes were isolated after 24 h and analyzed for (a) luciferase activity by luciferase assay (b) GFP and Luc gene expression by real-time PCR. Balb/C mice were HTV injected with pCAG·GFP in 0.9% saline and hepatocytes were isolated after 24 h and analyzed for (c) eGFP gene expression by real-time PCR (d) transfection efficiency by FACS. (e) Liver samples were frozen and sectioned to image transfected hepatocytes under (i) differential interference contrast (DIC) illumination or (ii) blue light excitation (10x).
(TIF)

Figure S2 Flow cytometry analysis of *in vivo*-reprogrammed hepatocyte extracts. Balb/C mice HTV injected with 0.9% saline alone, 75 µg of pCX-OKS-2A and 75 µg pCX-cMyc in 0.9% saline, or 150 µg of pCAG-GFP in 0.9% saline. On days 1, 4, 9, 11 and 22, hepatocytes were isolated and stained for OCT3/4, SOX2 and NANOG.
(TIF)

Figure S3 NANOG immunofluorescence staining of different liver sections after HTV injection of reprogramming plasmids. Balb/C mice HTV injected with 75 µg of pCX-OKS-2A and 75 µg pCX-cMyc in 0.9% saline. On day 4, liver tissue was collected and frozen tissue sections were immunostained with an anti-NANOG antibody. Scale bars represent 100 µm.
(TIF)

Figure S4 The effect of HTV injection of plasmids on liver damage at early time points. Balb/C mice HTV injected with either 75 µg of pCX-OKS-2A and 75 µg pCX-cMyc in 0.9% saline or 0.9% saline only. On days 2, 4, 8, 12 sera were isolated and analyzed for the levels of liver enzymes.
(TIF)

Figure S5 Plasmid DNA maps used in this study. **(a)** pCX-OKS-2A, **(b)** pCX-c-Myc, **(c)** pCAG-GFP and **(d)** pGFP-Luc plasmids.
(TIF)

Table S1 Primer seqeunces used in this study.
(TIF)
