# Supplementary figures and images for: In Vivo Cell Reprogramming towards Pluripotency by Virus-Free Overexpression of Defined Factors
Source: PLoS One. 2013 Jan 23;8(1):e54754. doi: 10.1371/journal.pone.0054754 (PMC3552956; doi:10.1371/journal.pone.0054754)

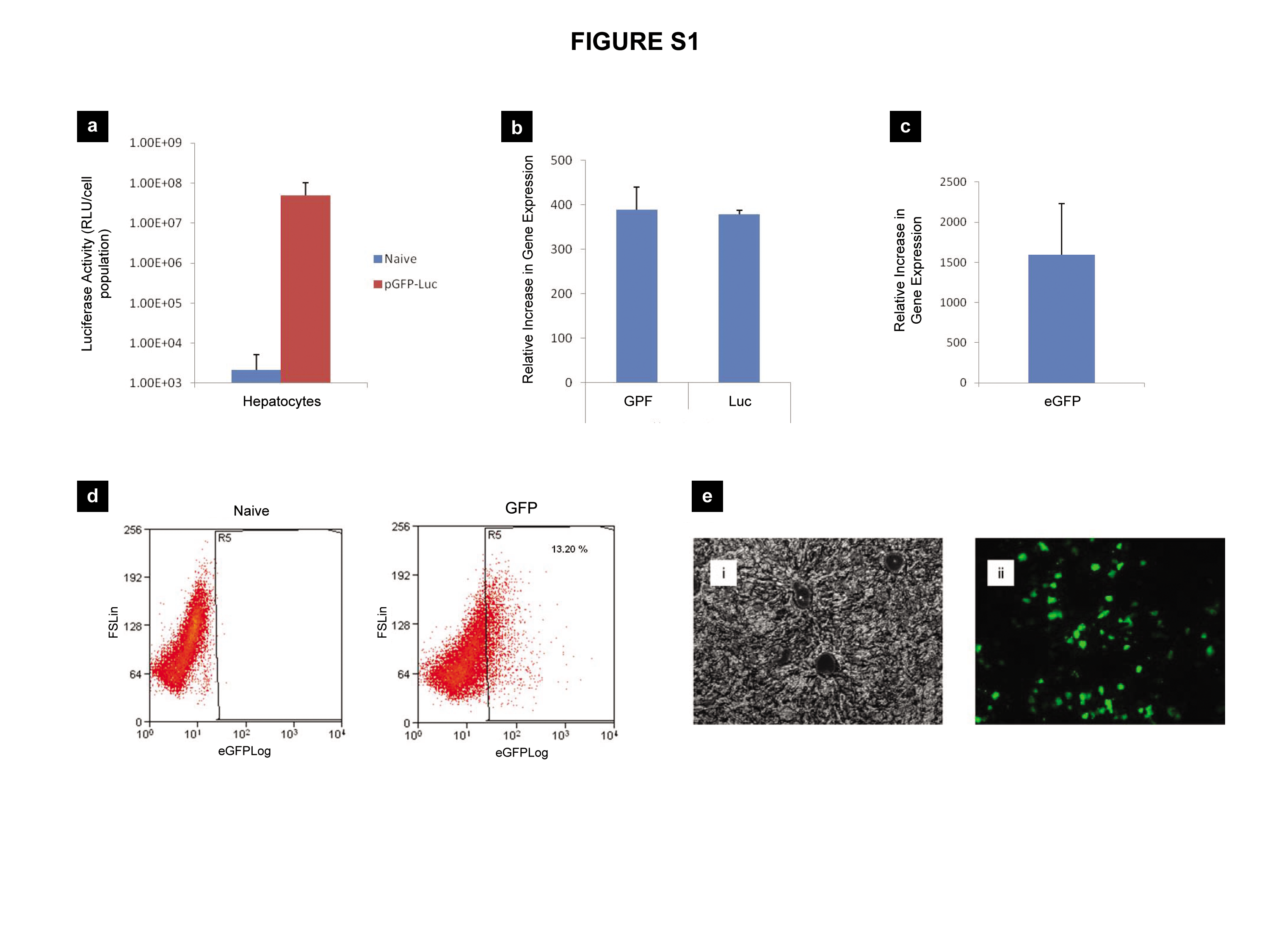

Supplement: Figure S1 — Transfection after HTV injection. Balb/C mice were HTV injected with pCMV·GFP-Luc in 0.9% saline and hepatocytes were isolated after 24 h and analyzed for (a) luciferase activity by luciferase assay (b) GFP and Luc gene expression by real-time PCR. Balb/C mice were HTV injected with pCAG·GFP in 0.9% saline and hepatocytes were isolated after 24 h and analyzed for (c) eGFP gene expression by real-time PCR (d) transfection efficiency by FACS. (e) Liver samples were frozen and sectioned to image transfected hepatocytes under (i) differential interference contrast (DIC) illumination or (ii) blue light excitation (10x). (TIF) [file pone.0054754.s001.tif]

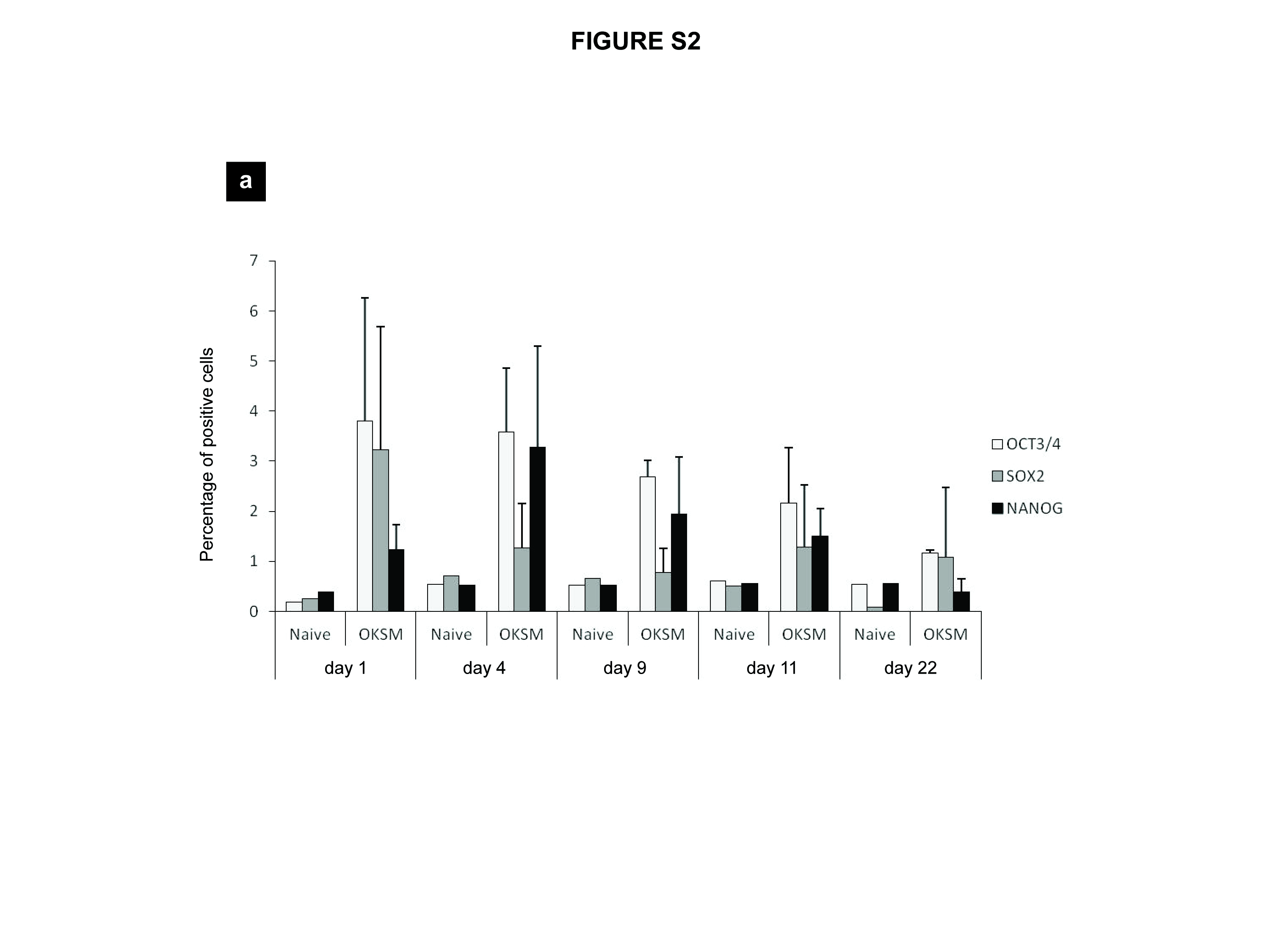

Supplement: Figure S2 — Flow cytometry analysis of in vivo-reprogrammed hepatocyte extracts. Balb/C mice HTV injected with 0.9% saline alone, 75 µg of pCX-OKS-2A and 75 µg pCX-cMyc in 0.9% saline, or 150 µg of pCAG-GFP in 0.9% saline. On days 1, 4, 9, 11 and 22, hepatocytes were isolated and stained for OCT3/4, SOX2 and NANOG. (TIF) [file pone.0054754.s002.tif]

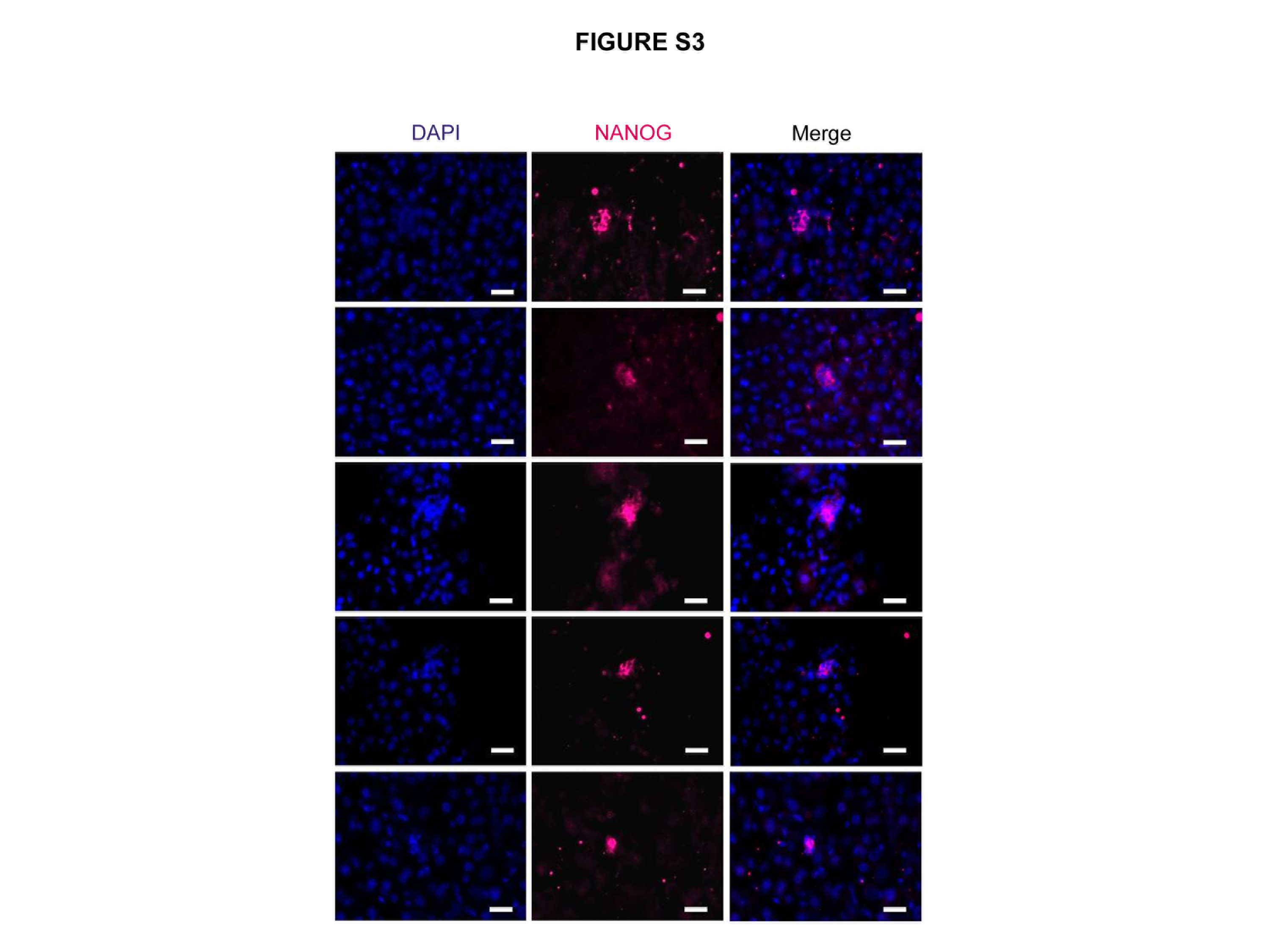

Supplement: Figure S3 — NANOG immunofluorescence staining of different liver sections after HTV injection of reprogramming plasmids. Balb/C mice HTV injected with 75 µg of pCX-OKS-2A and 75 µg pCX-cMyc in 0.9% saline. On day 4, liver tissue was collected and frozen tissue sections were immunostained with an anti-NANOG antibody. Scale bars represent 100 µm. (TIF) [file pone.0054754.s003.tif]

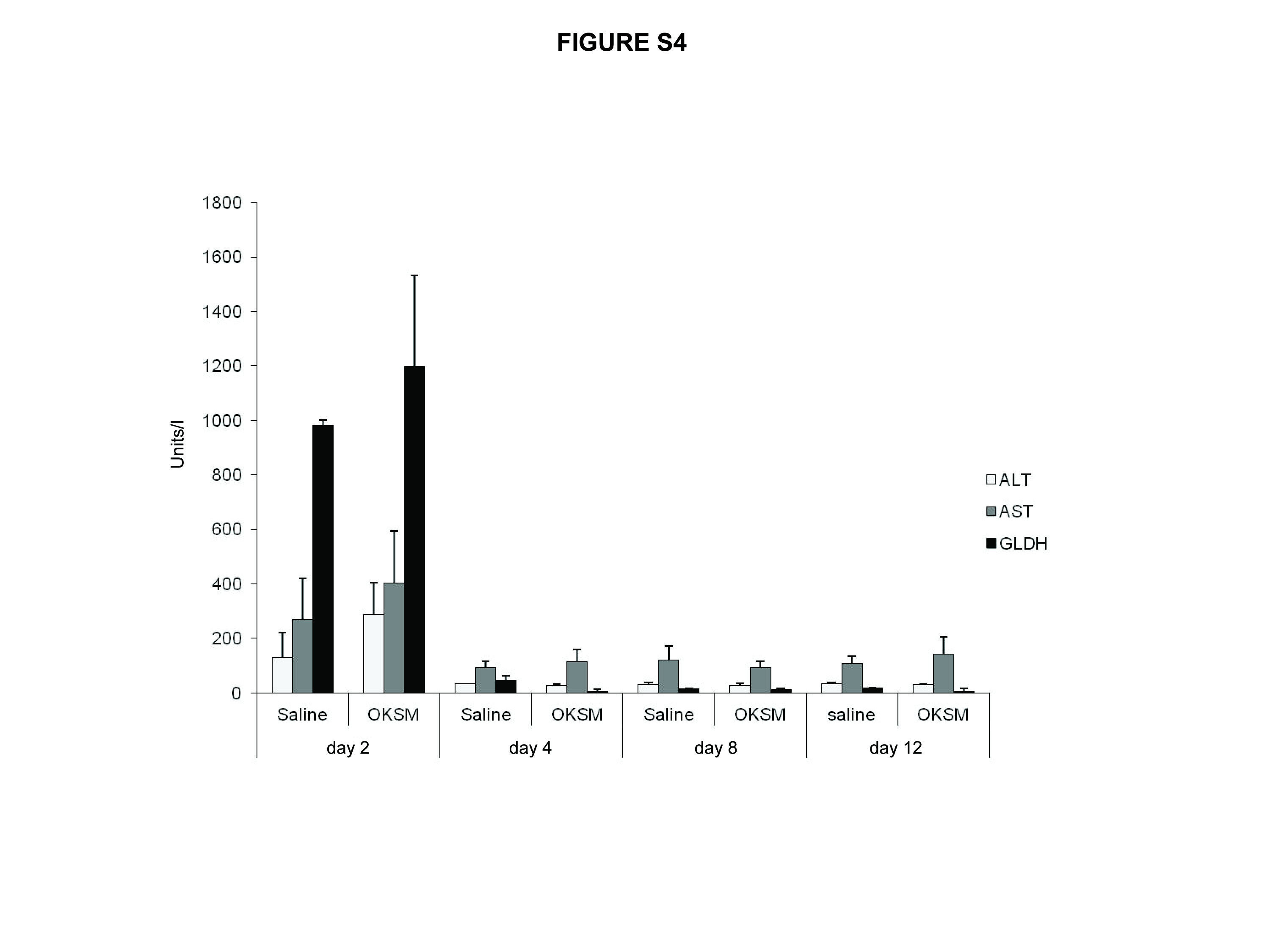

Supplement: Figure S4 — The effect of HTV injection of plasmids on liver damage at early time points. Balb/C mice HTV injected with either 75 µg of pCX-OKS-2A and 75 µg pCX-cMyc in 0.9% saline or 0.9% saline only. On days 2, 4, 8, 12 sera were isolated and analyzed for the levels of liver enzymes. (TIF) [file pone.0054754.s004.tif]

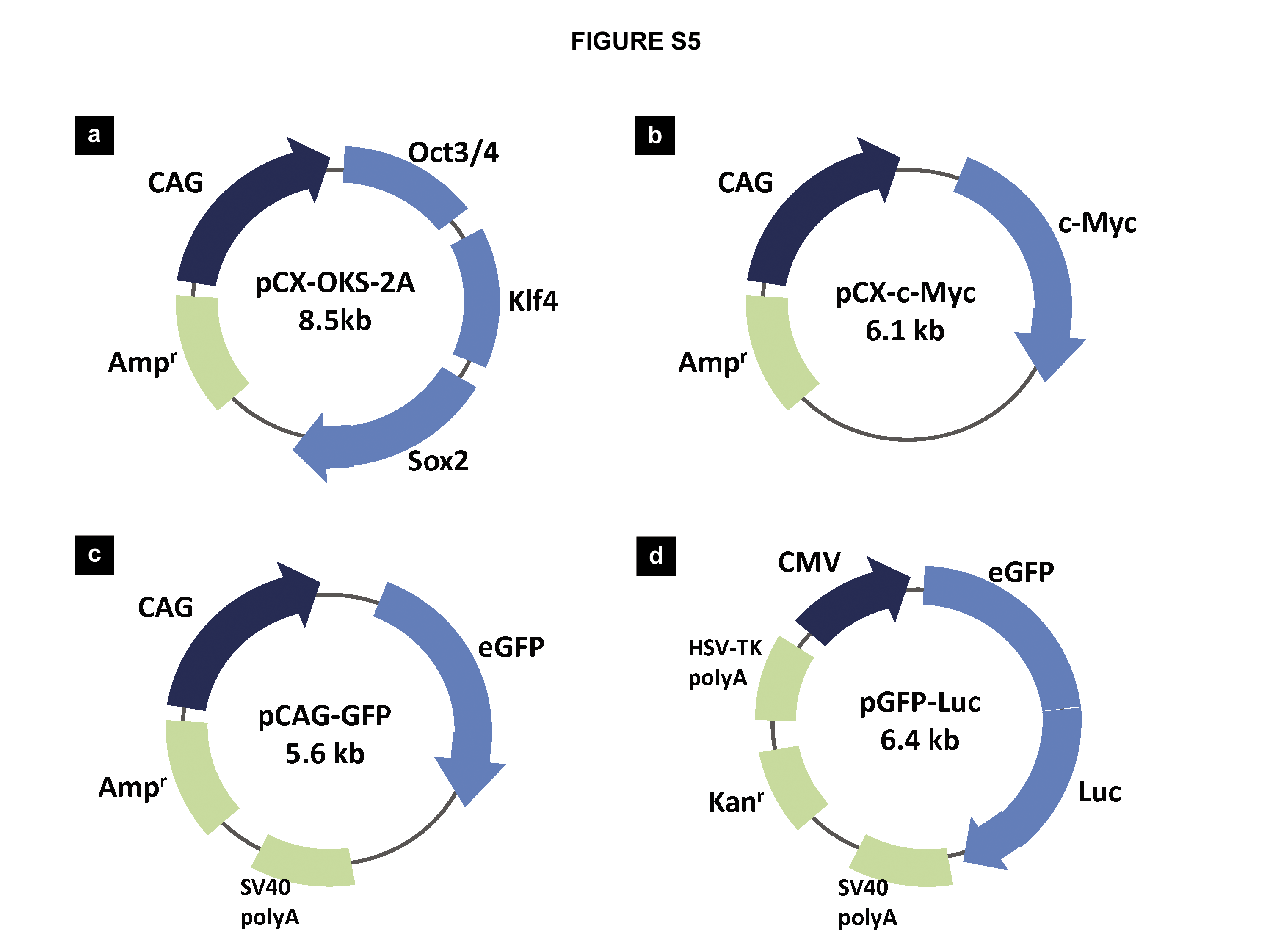

Supplement: Figure S5 — Plasmid DNA maps used in this study. (a) pCX-OKS-2A, (b) pCX-c-Myc, (c) pCAG-GFP and (d) pGFP-Luc plasmids. (TIF) [file pone.0054754.s005.tif]

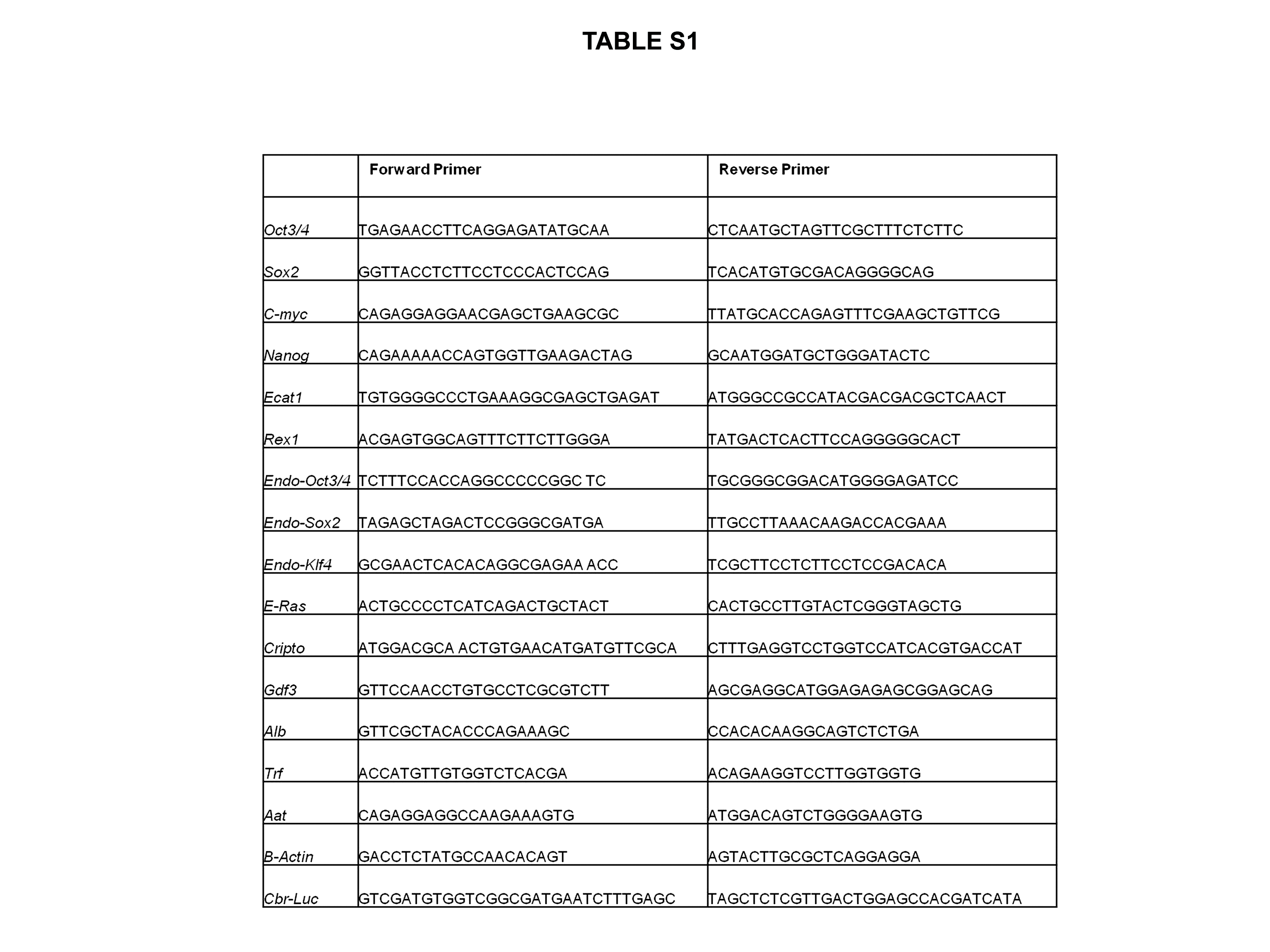

Supplement: Table S1 — Primer seqeunces used in this study. (TIF) [file pone.0054754.s006.tif]
